# Adult Sinonasal Rhabdomyosarcoma With Spinal Metastasis: A Case Report and Review of the Literature

**DOI:** 10.7759/cureus.25886

**Published:** 2022-06-12

**Authors:** Abdulaziz K Alaraifi, Raghad K Alsalamah, Abdulaziz A Alsalem, Abdul Latif Khan, Mohammed Elkrim

**Affiliations:** 1 Division of Otolaryngology, Head and Neck Surgery, Department of Surgery, King Abdulaziz Medical City, Ministry of National Guard Health Affairs, Riyadh, SAU; 2 College of Medicine, King Saud Bin Abdulaziz University for Health Sciences College of Medicine, Riyadh, SAU; 3 Department of Pathology and Laboratory Medicine, King Abdulaziz Medical City, Ministry of National Guard Health Affairs, Riyadh, SAU

**Keywords:** rhabdomyosarcoma (rms), tumor, adult, rhabdomyosarcoma, sinonasal

## Abstract

Rhabdomyosarcoma (RMS) is a malignant soft-tissue tumor mainly seen in the pediatric population. Here, we describe a case of an aggressive sinonasal RMS with distant metastasis in an adult patient. A 51-year-old male presented to the otolaryngology clinic with a unilateral painless neck mass and nasal obstruction. A flexible transnasal endoscope showed a huge fungating mass obstructing more than 80% of the right nasal cavity. A contrasted computed tomography (CT) scan of the paranasal sinuses showed an enhancing soft-tissue density mass involving the right nasal cavity. A biopsy revealed RMS, an embryonal variant. The patient responded well to chemoradiotherapy but later developed spinal metastasis and cord compression. He was admitted for palliative care but died due to cardiopulmonary arrest 10 months after diagnosis. A high index of clinical suspicion for malignancy is required in adult patients with unilateral nasal symptoms.

## Introduction

Rhabdomyosarcoma (RMS) is a malignant soft-tissue tumor usually seen in the pediatric population. It accounts for 5% of all pediatric malignancies and around 50% of soft-tissue sarcomas diagnosed in children [1]. On the other hand, RMS occurrence in adults is infrequent, representing less than 1% of adult malignancies and only 3% of soft-tissue tumors [2]. RMS originates frequently from the head and neck region, followed by the genitourinary tract and extremities [3,4].

Sinonasal RMS has an overall worse prognosis with a high mortality rate, mainly due to its usual presentation in advanced stages and its tendency for intracranial spread [1,5]. The presentation in late stages is usually due to its mild nonspecific symptoms, including intermittent epistaxis, rhinorrhea, and nasal obstruction [6]. Multimodality treatment, including chemotherapy and radiotherapy with or without surgical resection, is recommended for sinonasal RMS in children [7]. However, there is limited data in the literature on the management and outcome of sinonasal RMS in adults. We report a case of an adult patient with sinonasal RMS of an embryonal variant that only survived 10 months after diagnosis despite treatment with chemoradiotherapy.

## Case presentation

**Figure 1:**
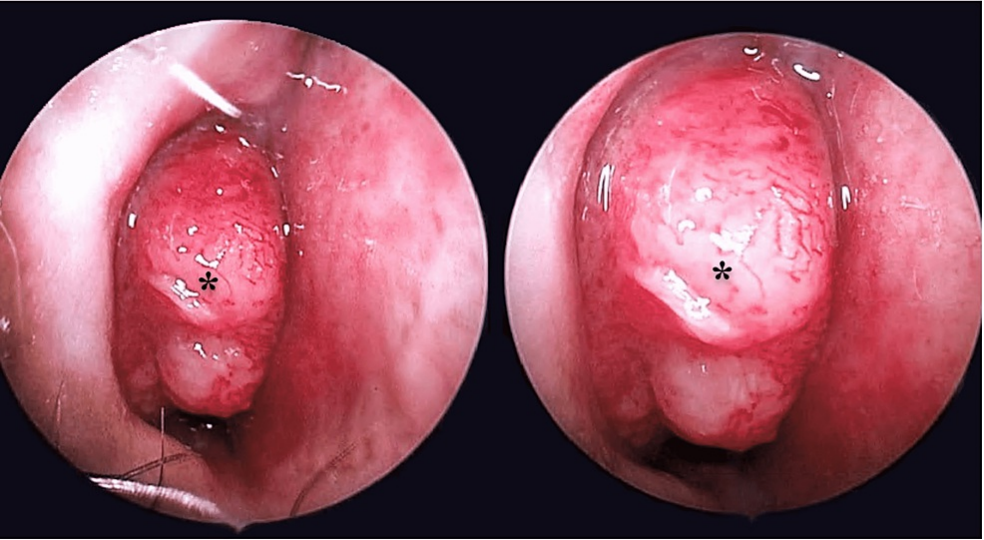
A large fungating mass obstructing more than 80% of the right nasal cavity, adherent to the lateral nasal wall and extending superiorly (asterisk).

A 51-year-old male presented to the otolaryngology clinic with a painless right-sided nonprogressive neck mass and a right-sided nasal obstruction for a one-month duration. He did not have other sinonasal symptoms (e.g., epistaxis or rhinorrhea), compressive symptoms (e.g., dysphagia, dyspnea, or dysphonia), and neither fever nor constitutional symptoms. The patient’s medical history was only significant for diabetes mellitus and smoking. Physical examination revealed a palpable, hard, non-tender level II cervical lymph node on the right side measuring around 3.0 × 1.0 cm. Flexible transnasal endoscope showed a huge fungating mass obstructing more than 80% of the right nasal cavity, adherent to the lateral nasal wall and extending superiorly (Figure 1). The left nasal cavity was patent with no masses. Examination of the nasopharynx, oropharynx, and hypopharynx was unremarkable. Cranial nerves were all intact as well.

**Figure 2:**
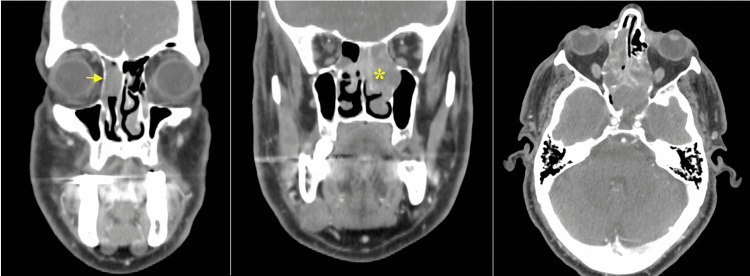
A contrasted CT scan of the paranasal sinuses showing a faintly enhancing soft-tissue mass in the right nasal cavity (arrow) with extension to the left nasal cavity, right frontal sinus, bilateral ethmoidal sinuses, left maxillary wall, and left sphenoid (asterisk).

**Figure 3:**
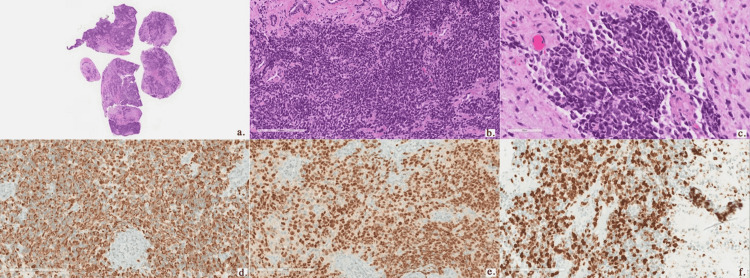
Multiple biopsies of the mass show a diffusely infiltrating, high-grade, undifferentiated malignant round blue cell tumor (Hematoxylin and Eosin; a-c). The tumor cells are positive for desmin (d) and myogenin (e) and have a high Ki67 proliferation index (f).

**Figure 4:**
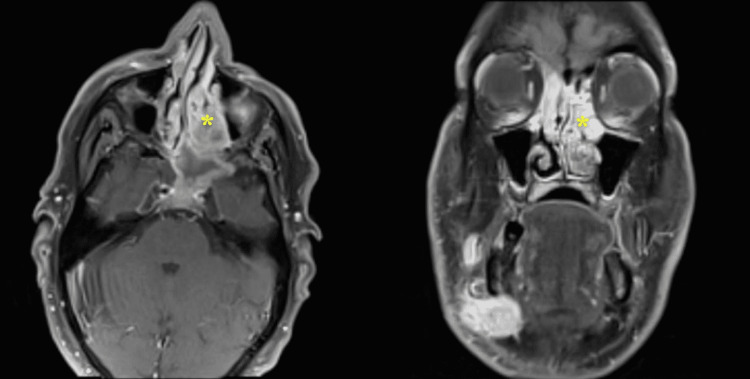
Axial and coronal MRI with contrast (T1 sequences) of the paranasal sinuses showing a homogeneous enhancing infiltrating soft-tissue mass (asterisk) with no orbital or intracranial extension.

A contrasted computed tomography (CT) scan of the paranasal sinuses and neck showed a faintly enhancing soft-tissue mass in the right nasal cavity with extension to the left nasal cavity, right frontal sinus, bilateral ethmoidal sinuses, left maxillary wall and left sphenoid (Figure 2). Multilevel bilateral partially enhanced enlarged cervical lymph nodes measuring as follows were found in the highest dimension: right level IIa and level IIb (3 cm and 3.5 cm, respectively), left level IIa (2.5 cm), and left supraclavicular lymph node (1.4 cm). A contrasted chest CT scan revealed bilateral suspicious pulmonary nodules, measuring 7 mm and 5 mm in the left and right lower lobes, respectively. The patient underwent examination of the nasal cavity and nasopharynx under local anesthesia in the operating room with multiple biopsies from the right nasal cavity mass. The biopsies showed a poorly differentiated malignant round blue cell neoplasm consisting of sheets and nests of pleomorphic neoplastic cells containing hyperchromatic and eccentric nuclei. Immunohistochemical staining showed positivity for desmin, myogenin, and CD-99, with a high Ki67 proliferation index (Figure 3). The epithelial, neuroendocrine, melanoma, and lymphoma markers were negative. These findings were consistent with RMS, favoring the embryonal subtype. Ultrasound-guided fine-needle aspiration (FNA) from the right level IIa cervical lymph node showed a malignant small round blue cell tumor, consistent with metastatic RMS. A magnetic resonance imaging (MRI) of the paranasal sinuses showed no orbital or intracranial extension (Figure 4). A diagnosis of embryonal RMS with the clinical stage of T2aN1M0 was established.

**Figure 5:**
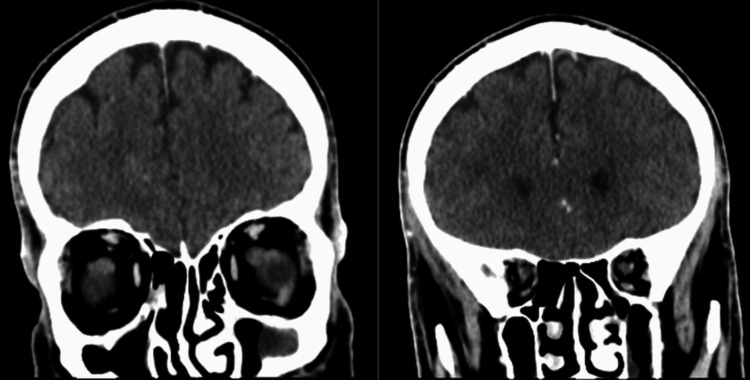
A contrasted CT scan of the paranasal sinus following completion of chemoradiotherapy showing complete resolution of the sinonasal mass.

After conducting a tumor board meeting, the patient was referred to the oncology department and received two cycles of multiagent chemotherapy plus radiotherapy. Following the completion of treatment, a repeated CT scan showed a marked interval improvement and shrinkage of the tumor and previously enlarged lymph nodes (Figure 5). One month later, the patient developed spinal metastases and cord compression. The patient underwent surgical decompression and fixation of T8 and T11, followed by palliative radiotherapy to the thoracic spine. Due to distant metastasis, the patient received palliative chemotherapy as well. The patient was then admitted to the hospital for palliative care as his general status deteriorated with decreased oral intake, increased confusion, and agitation. During the admission, the patient died due to cardiopulmonary arrest. Table 1 demonstrates the chronological events of the case.

**Table 1:** Chronological events of the case. TNM: tumor, nodes, and metastases

Day	Event
0	Initial presentation.
5	Chest, neck, and sinus contrasted computed tomography (CT).
7	Endoscopic biopsy and fine-needle aspiration (FNA).
11	Sinonasal magnetic resonance imaging (MRI).
14	Diagnosis of embryonal rhabdomyosarcoma (T2aN1M0).
32	Treatment initiation.
131	Treatment course completion.
155	Follow-up imaging showed interval improvement and shrinkage of the tumor.
193	Spinal metastases and cord compression were diagnosed.
204	Surgical decompression and fixation.
214	Started palliative radiotherapy on the thoracic spine.
252	Palliative chemotherapy initiation.
291	Palliative care admission.
314	Death due to cardiopulmonary arrest.

## Discussion

Sinonasal RMS has an incidence of 0.034 per 100,000 cases, predominantly reported in the pediatric population [8]. In adults, sinonasal RMS is rare and more commonly seen in patients younger than 35 years old, whereas our patient was a 51-year-old at the time of the diagnosis [9,10]. RMS in adults differs from pediatrics in presentation, histopathology, prognosis, and response to treatment. RMS originates from mesenchymal cells and is included among small blue round cell tumors [11]. It is classified into four histological variants based on unique morphologic characteristics. These include embryonal, alveolar, pleomorphic, and sclerosing subtypes [12]. The most common variant is embryonal type, representing around 60% of all cases with childhood predominance. The alveolar variant is the most common form in adults, with a worse prognosis than the embryonal subtype [13].

The head and neck region represents the most common site for the primary tumor accounting for one-third of the cases, followed by the genitourinary tract and extremities [3,4]. The paranasal sinuses represent 10-15% of RMS tumors in the head and neck region. Maxillary and ethmoid sinuses are the most common locations for sinonasal RMS [1]. The other sites for RMS in the head and neck region include orbital and non-orbital sites, such as the mouth, neck, face, scalp, and larynx [3,4]. The presentation of RMS varies from asymptomatic mass to signs and symptoms related to the primary site. Patients with sinonasal RMS usually present in advanced stages with mild symptoms, such as nasal obstruction, intermittent epistaxis, or unilateral rhinorrhea [6]. Patients may also present with an asymptomatic mass in the neck, as reported in our case.

Imaging studies for the primary tumor include CT scan and MRI. CT scan helps determine the size of the tumor, bone erosion, and metastatic evaluation. On the other hand, MRI evaluates orbital and intracranial involvement in case of suspicion. However, the diagnosis is established based on histopathological characteristics [3].

Studies on the management of sinonasal RMS in adults are limited, mainly due to the rare nature of the disease and its limited prevalence in adults. The current standard of care is a multimodality treatment that includes chemotherapy and radiotherapy [3,7]. Surgical resection is valid if it does not cause significant functional or cosmetic morbidity [11]. The surgeon must weigh the benefits and risks of such intervention. A wide complete surgical resection with adequate margins is usually impossible in sinonasal RMS due to its proximity to the orbit and skull base. However, the impact of surgical resection on the outcome of the disease is not well-studied in the literature.

Multiple factors influence the prognosis and course of the disease, including age, anatomical site, tumor size, histological subtype, and the presence of regional or distant metastasis [14,15]. Sinonasal RMS has a poor prognosis with a high incidence of distant metastasis in adults compared to the pediatric population, even after treatment, as reported in our case. Lung and bone are the common sites of metastasis. Distant metastasis occurs in more than 60% of adult patients at the time of diagnosis. This is especially true for sinonasal RMS in adults, where the 5-year survival rate is 8% [16].

## Conclusions

RMS is a rare tumor in adults with poor outcomes compared to the pediatric population. Due to its usual presentation in advanced stages, sinonasal RMS has a high mortality rate. Multimodality treatment, including chemotherapy and radiotherapy, is the preferred plan for the treatment with multidisciplinary team care to optimize patient health and outcomes. A high index of clinical suspicion for malignancy is required in adult patients with unilateral nasal symptoms.
